# Impaired Conflict Monitoring to Food Cues in Women Who Binge Eat

**DOI:** 10.3389/fpsyg.2018.02585

**Published:** 2018-12-17

**Authors:** Zhenyong Lyu, Panpan Zheng, Songkai Lu, Mingzhi Qin

**Affiliations:** ^1^School of Education Science, Xinyang Normal University, Xinyang, China; ^2^Key Laboratory of Cognition and Personality, Southwest University, Chongqing, China

**Keywords:** binge eating, flanker task, conflict monitoring, motor impulsivity, food cue

## Abstract

Previous research demonstrated the associations between cognitive biases toward food cues and binge eating (BE) behavior. To determine the characteristics of conflict monitoring to food cues in women who binge eat and non-eating disordered controls, a flanker task featured high-caloric food and low-caloric food images was used to examine conflict monitoring with measures of accuracy and reaction time. Women who binge eat displayed longer reaction times (RTs) to incongruent trials (i.e., flanked by pictures from the different category) than to congruent trials (i.e., flanked by pictures from the same category), while controls showed no such difference. This finding demonstrated women who binge eat displayed a general flanker effect toward food-related stimuli compared to controls. Faster reaction times in response to high-caloric food images disturbed by low-caloric food images predicted lower self-reported motor impulsiveness in the women who binge eat, but not in controls. These data suggest a relative conflict monitoring deficit in women with BE pathology.

## Introduction

Binge eating (BE) refers to consumption of an objectively large amount of food within a short period of time, accompanied by a perceived loss of control over eating. Frequent BE is a core diagnostic feature of clinical eating disorders, and persistent symptoms increase risk for Binge-Eating Disorder (BED) according to the 5th edition of the Diagnostic and Statistical Manual of Mental Disorders (American Psychiatric Association, 2013). Diagnosis of Bulimia Nervosa (BN) requires regular binge eating and recurrent compensatory responses to such episodes. BE is prevalent among people seeking to lose or maintain weight (Coker et al., 2015), and affects up to 40% of college-age women in United States (Saules et al., 2009). Furthermore, binge eating behavior is increasing in China (Chen and Jackson, 2008; Tong et al., 2014), and over one third of Chinese adolescents and young adults have reported BE (Chen and Jackson, 2008). Indeed, individuals with binge eating often experience depression, interpersonal problems and reduced quality of life (Ambwani et al., 2015; Rosenbaum and White, 2015).

Cognitive models of obesity suggest reduced inhibitory control is an important causal and maintenance factor in obesity and eating disorders (Jansen et al., 2015). Recent studies have linked cognitive impairment toward food-related stimuli to disordered eating behavior, such as binge eating (Mobbs et al., 2011; Svaldi et al., 2014a,b; Manasse et al., 2016; Kollei et al., 2018; Leehr et al., 2018). An essential component of cognitive control is conflict monitoring (Luna et al., 2015). Conflict monitoring refers to the ability to detect information conflict and reactively increase cognitive control recruitment (Botvinick et al., 2001; Teubner-Rhodes et al., 2016). Poor conflict monitoring might contribute to the frequent initiation of eating episodes, which is associated with obesity. Moreover, poor ability to inhibit an already-initiated motor response (e.g., eating) might contribute to the development of BE (Manasse et al., 2016). However, the knowledge about the conflict monitoring in those with BE is still scarce.

The Stroop color-word interference task has been used as a measure of conflict monitoring and response selection among individuals with eating disorders (Duchesne et al., 2010; Galioto et al., 2012; Balodis et al., 2013; Kittel et al., 2017), though mixed findings have been observed between individuals with BE/BED and controls. For example, Kittel et al. (2017) investigated the conflict monitoring in obese individuals with BED as compared to obese individuals without BED and normal weight participants using a Stroop color-word interference task. They found obese individuals with BED and without BED performed worse than normal weight participants, while the obese individuals with BED and obese individuals without BED did not differ (Kittel et al., 2017). The results hint at general impaired conflict monitoring in youth with BED and obesity in comparison with the normal weight group. However null effects are also existed (Duchesne et al., 2010; Galioto et al., 2012). One potential explanation for the mixed findings is that neutral stimuli (e.g., letters of the alphabet) are probably less rewarding and emotionally arousing compared to food-related images or actual food stimuli, reducing the ecological validity of Stroop tasks (Berner et al., 2017). A meta-analysis of the Stroop task on eating disorders demonstrated that individuals with binge/purge eating disorders showed more cognitive control deficits when food-specific stimuli were used compared to controls (Wu et al., 2014). Cognitive control deficits in those with BE may be especially pronounced when relevant stimuli are used, i.e., food cues (Manasse et al., 2016). In a functional magnetic resonance imaging study, Lee et al. (2017) modified the Stroop match-to-sample task using two different conditions, food-related condition and neutral condition, to investigate the effect of food stimuli on cognitive controls in individuals with eating disorder. The cues with different colors consisted of three letters (i.e., XXX). Participants matched the color of the cue (i.e., “XXX”) to the written color of a Stroop word target or to the color that the Stroop word means, which appeared after an interference stimulus (e.g., food-related and neutral pictures). No significant differences were observed in accuracy and reaction time between the BED group and control group (Lee et al., 2017). However, BED group demonstrated stronger activations in the ventral striatum in response to food images compared to control group, indicating BED patients exhibited increased reward sensitivity without inhibitory control (Lee et al., 2017). Together, these findings suggest that individuals with BE/BED are likely to show conflict monitoring deficits though mixed findings have been existed.

The flanker task is another paradigm that has been widely used to assess the ability to inhibit distraction and adapt to conflict (Eriksen and Eriksen, 1974). The Stroop-like tasks elicited responses to non-symbolic information (e.g., color of a letter), whereas flanker task elicited responses to symbolic information (e.g., arrow meaning) (Freitas et al., 2007). In the flanker task, participants respond to the “target” stimulus displayed in the middle of the screen, which are flanked on each side by either the same as the target (i.e., congruent, > > > > > ) or different from the target (i.e., incongruent, > > < > >). Participants are instructed to attend to the target stimuli and ignore the non-target stimuli. Because of the interference elicited by the incongruent non-target stimuli, participants generally required longer reaction times to incongruent trials than to congruent trials (Eriksen and Eriksen, 1974; Coles et al., 1985). This “flanker effect” phenomenon, that is, slower responses to incongruent trials, provides an index of the conflict monitoring. Due to the lower levels of cognitive control being successfully applied, the flanker effect will be larger, which indicates a greater conflict monitoring deficit (Husted et al., 2016).

Conflict monitoring to food-related stimuli has been examined using flanker task in previous research (Forestell et al., 2012; Meule et al., 2012; Husted et al., 2016). For example, using a flanker task involved high caloric food-cues and neutral pictures, Meule et al. (2012) found restrained eaters responded faster to high-calorie food targets as compared to neutral targets than unrestrained eaters, suggesting a low self-regulatory ability in restrained eaters (Meule et al., 2012).

Based on the literature outlined above, a food-related flanker task with high- and low-calorie food images was introduced to examine the interference from food stimuli on cognitive control in women who binge eat. We hypothesized that the women who binge eat would show greater deficits in response conflict than the control group on both stimulus types of the flanker task.

## Materials and Methods

### Participants

Thirty-one undergraduate women with BE and 33 healthy control participants were recruited from a large Chinese university. Average age was *M* = 20.91 years (*SD* = 1.52), and the mean BMI was *M* = 20.68 kg/m^2^ (*SD* = 2.66). All women who binge eat reported at least one binge eating episode per week over the past 3 months (*M* = 2.52, *SD* = 2.12, range: 1–12) on the Eating Disorder Diagnostic Scale (EDDS; Stice et al., 2000) as well as an absence of compensatory behavior following BE episodes. Healthy control participants reported no current nor past eating disorder according to DSM-5 criteria. In order to standardize hunger levels, all participants were instructed to refrain from eating and drinking caffeinated beverages for 12 h before the study which occurred between 8 and 11 am the next day.

### Materials

Forty-eight colorful images each of high-calorie foods (e.g., hamburger, doughnuts, and fried chicken wings) and low-calorie foods (e.g., tomatoes, carrots) were used. All images were taken from a set previously used in our studies (Lyu and Jackson, 2016; Lyu et al., 2016, 2017), and edited to be homogeneous with respect to background color.

### Procedure

This study was performed in accordance with the guidelines of the International Committee of Medical Journal Editors. The study was approved by the Human Research Ethics Committee of the Xinyang Normal University. Potential volunteers were recruited via the campus electronic bulletin board system and flyers. Screening materials including demographic items and the Eating Disorder Diagnostic Scale (EDDS) were completed (Stice et al., 2000). Participants arrived at the lab individually for their scheduled appointment. Upon arriving, volunteers were informed of the general research focus (i.e., attention toward different kinds of food images) and gave the informed consent prior to their participation. Subjective feeling of hunger were assessed individually on a visual analog scale from 1 to 9 (1: not hungry at all; 9: very hungry).

The flanker task procedures were programmed using E-Prime 2.0. The flanker task comes from prior food flanker studies (Forestell et al., 2012; Meule et al., 2012; Husted et al., 2016). In the food flanker task, the central targets were pictures of either high-calorie foods or low-calorie foods images, which were flanked by pictures either from the same category (congruent condition) or distractors from the other category (incongruent condition). Participants were instructed to respond to the centrally presented picture (i.e., target) as quickly and accurately as possible by pressing a left or right button to indicate whether the target was a high-calorie food or a low-calorie food item (mapping was counterbalanced across participants). A 2 min practice session commenced first to ensure that participants become familiar with the procedure before the formal study. Each trial consisted of a prestimulus baseline during which a fixation cross was presented in the middle of the screen for 1000 ms. This was followed by a stimulus array contained one target picture and two pictures on either side of the target (i.e., flankers) followed by a blank screen. After the target appeared in the center of the screen, participants were instructed to respond as quickly as possible. The target remained on the screen until a response was detected or 1500 ms passed. Pictures for flankers and targets were randomly drawn from the same food image set of high or low calorie. In the congruent condition, target and flanker pictures were the homogeneous: (1) all the pictures were high-calorie foods (HHH); (2) all the pictures were low-calorie foods (LLL). In the incongruent condition, target and flanker pictures differed: (3) the target was a high-calorie food picture and the flankers were two low-calorie food pictures (LHL); (4) the target was a low-calorie food picture and the flankers were two high-calorie food pictures (HLH). Inter-trial intervals varied randomly from 1000 to 3000 ms to avoid time conditioning. The task included 192 trials in total divided in three blocks.

After the flanker task, the self-report scales described below, except the EDDS, were completed in a quiet room. Finally, participants were required to make a guess regarding the main research purpose, and then were debriefed about the study hypotheses. No participants identified BE or response conflict as foci of the experiment. Participants received 20 yuan as compensation.

### Questionnaires

#### Eating Disorder Diagnostic Scale (EDDS; Stice et al., 2000)

The EDDS is a 22-item self-report scale that based on Diagnostic and Statistical Manual-IV criteria for Anorexia Nervosa (AN), BN, and BED. In the present study, EDDS was used to identify individuals with BE as well as to rule an eating disorder diagnosis among the control group members. An overall composite calculated from the sum of z-scores of the first 18 items also provided a symptom severity rating. The scale has satisfactory test–retest reliability, a high level of internal consistency, and excellent concordance with diagnoses based on structured interviews and other self-report measures of eating disturbances (Stice et al., 2000, 2004). The Chinese version of the EDDS also has satisfactory reliability and validity in samples of mainland Chinese adolescents and young adults (Jackson and Chen, 2014, 2015). The consistency was *α =* 0.76 in this sample.

#### Barratt Impulsiveness Scale-Chinese (BIS-C; Li et al., 2011)

The BIS-C, consisting of attentional, motor, and non-planning impulsiveness subscales, was used to assess rash-spontaneous behavior (Lyu et al., 2016, 2017). BIS-C has satisfactory psychometrics among Chinese undergraduates (Li et al., 2011). For this study, the internal consistency of the overall BIS-C was *α* = 0.74. Alphas values were acceptable for the three subscales (αs ≥ 0.71).

#### Uncontrolled Eating Scale (UES; Karlsson et al., 2000)

The binge eating level was assessed by summing seven items from the Three-Factor Eating Questionnaire-R18 in the formal study. The questionnaire has been found to have sound reliability and validity (Anglé et al., 2009; Lyu et al., 2017). Its alpha value was 0.84 in the current study.

#### Data Analyses

Data were analyzed with IBM SPSS Statistics 20.0. Group differences on demographics, impulsivity, and uncontrolled eating were assessed via *t*-tests. A 2 (Group: binge eating versus non-binge eating) × 2 (Food Type: high-calorie food versus low-calorie food) × 2 (Flanker: congruent versus incongruent) ANOVA was performed to assess accuracy and RT differences. Individual trials on the flanker task with errors and response times more than three standard deviations above or below the mean (i.e., 7% of trials) were excluded from analysis (Forestell et al., 2012). Mean accuracy and reaction times were calculated for each of the 3-picture array combinations (i.e., HHH, LLL, LHL, and HLH) for each participant. Mauchly’s test of sphericity was violated in ANOVA analyses; thus, Greenhouse-Geisser corrections were used to reduce risk for Type I errors.

## Results

### Group Differences on Demographics and Characteristics

As show in Table 1, women who binge eat displayed higher levels of motor impulsiveness and uncontrolled eating relative to controls. However, no group differences were observed on age, BMI, year in university, or hunger ratings.

**Table 1:** Characteristics of binge-eating group versus control group (M ± SE).

	Binge eating (*n* = 31)	Control (*n* = 33)	*t*	Cohen’s *d*
Age	20.00 (0.66)	21.15 (0.26)	-1.67	-0.41
Body mass index	21.03 (0.50)	20.36 (0.44)	1.01	0.25
Year in university	2.10 (0.16)	2.55 (0.17)	-1.91	-0.48
Hunger rating	6.13 (0.34)	6.79 (0.22)	-1.64	-0.41
EDDS (z-scores)	0.50 (0.16)	-0.47 (0.15)	-4.45***	1.10
BIS-C total score	96.06 (1.39)	92.79 (1.87)	1.39	0.35
BIS-C (motor impulsiveness)	29.32 (0.83)	25.58 (1.42)	2.24*	0.56
BIS-C (attention impulsiveness)	34.55 (0.68)	34.06 (0.75)	0.48	0.12
BIS-C (non-planning impulsiveness)	32.19 (0.80)	33.15 (1.11)	-0.69	-0.17
Uncontrolled Eating Scale	23.65 (0.50)	18.24 (0.74)	5.55***	1.40

*EDDS, Eating Disorder Diagnostic Scale; BIS-C, Barratt Impulsiveness Scale-Chinese; ^∗^ < 0.05; ^∗∗∗^ < 0.001.*

### Differences in Accuracy

The repeated measures ANOVA performed on accuracy revealed an interaction effect of Food Type × Flanker, *F*(1, 62) = 14.42, *p* < 0.001, $\eta_{p}^{2}$ = 0.19. Simple effects analyses indicated that a better performance of judgment was observed for congruent trials (*M* = 0.94, *SE* = 0.02) compared to incongruent trials (*M* = 0.91, *SE* = 0.02) with low-calorie food images as targets (*p* = 0.01), while a better performance of judgment was shown for incongruent trials (*M* = 0.94, *SE* = 0.01) than congruent ones (*M* = 0.92, *SE* = 0.02) with high-calorie food images as targets (*p* = 0.01). No other main effect or interaction effect was significant (*p’s* > 0.05).

### Differences in Reaction Time (RT)

A main effect for Food Type, *F*(1, 62) = 5.16, *p* = 0.02, $\eta_{p}^{2}$ = 0.20, indicated the sample responded more quickly to low-calorie food compared to high-calorie food images (*M* = 726.27 ms versus *M* = 739.99 ms, *p* = 0.03). As expected, the main effect for Flanker, *F*(1, 62) = 14.36, *p* < 0.001, $\eta_{p}^{2}$ = 0.19, evidenced a general flanker effect: congruent trials elicited significantly shorter RTs in the entire sample (*M* = 727.28 ms versus *M* = 738.98 ms, *p* < 0.001).

**FIGURE 1:**
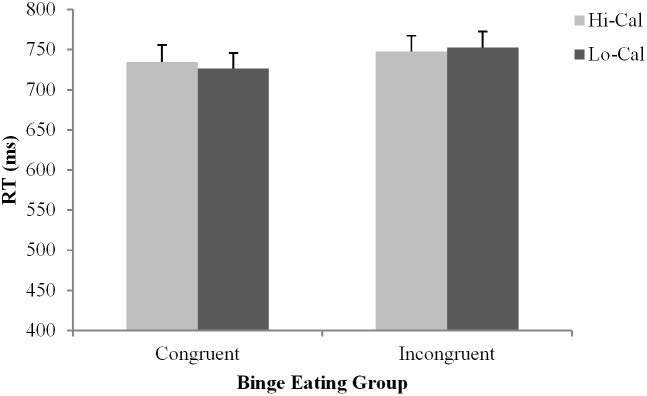
Reaction time data for women with binge eating. Bars represent standard error of the mean (SE). Hi-Cal, High-calorie; Lo-Cal, Low-calorie.

**FIGURE 2:**
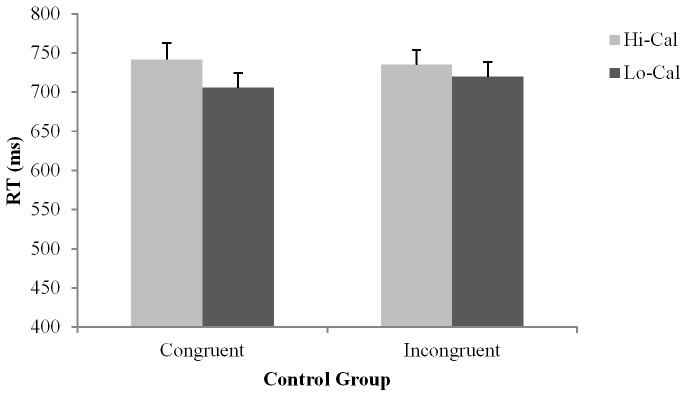
Reaction time data for controls. Bars represent standard error of the mean (SE). Hi-Cal, High-calorie; Lo-Cal, Low-calorie.

Simple effects analyses of the Group × Flank interaction, *F*(1, 62) = 6.69, *p* = 0.01, $\eta_{p}^{2}$ = 0.10, indicated women who binge eat displayed a significant flanker effect (*p* < 0.001), while no such effect was observed in the controls (*p* = 0.39) (see Figures 1, 2). The Food Type × Flanker interaction, *F*(1, 62) = 8.35, *p* = 0.01, $\eta_{p}^{2}$ = 0.12, indicated a strong flanker effect to low-calorie food images (*p* < 0.001), but not to high-calorie food images (*p* = 0.45). Furthermore, shorter RTs were observed to low-calorie food images compared to high-calorie food images in congruent trials (*M* = 716.16 ms versus *M* = 738.39 ms, *p* = 0.01), but no such difference was found in incongruent trials (*M* = 736.37 ms versus *M* = 741.58 ms, *p* = 0.43). For the Food Type × Group interaction, *F*(1, 62) = 4.11, *p* = 0.04, $\eta_{p}^{2}$ = 0.06, controls displayed shorter RTs to low-calorie food images than to high-calorie food images (*M* = 712.68 ms versus *M* = 738.65 ms, *p* = 0.01), but no such difference was found in women who binge eat (*M* = 741.32 ms versus *M* = 739.85 ms, *p* = 0.87). No other differences were observed significant.

To disentangle significant relations between flank task responses and self-reported measures, supplementary correlation analyses were performed within group. Women who binge eat displayed negative correlations between self-reported motor impulsiveness score and accuracy rates in different conditions (HHH: *r* = -0.41, *p* = 0.02; LHL: *r* = -0.46, *p* = 0.01; LLL: *r* = -0.47, *p* = 0.01), though a marginally significant correlation was observed (HLH: *r* = -0.35, *p* = 0.05). Conversely, control group members showed no such correlations between self-reported motor impulsiveness and accuracy rates for HHH (*r* = 0.01, *p* = 0.99), LHL (*r* = -0.01, *p* = 0.98), LLL (*r* = -0.01, *p* = 0.99), and HLH (*r* = -0.03, *p* = 0.88). Shorter RTs in response to high-caloric food images disturbed by low-caloric food images (i.e., LHL) were related to higher motor impulsiveness in women who binge eat (*r* = 0.44, *p* = 0.01) but not in controls (*r* = 0.03, *p* = 0.87). No other correlations were observed significant.

## Discussion

The present study investigated the conflict monitoring in women who binge eat during exposure to food cues. To the best of our knowledge, this is the first study that used food-related flanker task to assess conflict monitoring in women with BE and controls. Regarding the central research focus, women with BE show greater deficits in response conflict on the food-related flanker task, which support the main hypothesis.

Compared to controls, participants with BE reported higher trait impulsivity and uncontrolled eating, in line with the criterion of BE and previous research (Schag et al., 2013; Svaldi et al., 2014a), especially on motor impulsivity (Nasser et al., 2004; Galanti et al., 2007; Lyu et al., 2016, 2017). The motor impulsivity is defined as acting without thinking (Stanford et al., 2009), and could be used to distinguish eating disorder subtypes (Tillman and Wiens, 2011). Previous research have found the motor impulsivity was positively correlated with test meal intake and mood rated before consuming the test meal (Nasser et al., 2004; Galanti et al., 2007), and positively predicted binge eating and general eating pathology (Meule and Platte, 2015). Motor impulsivity involves the ability to suppress a prepotent yet inapposite motor response (Chamberlain and Sahakian, 2007). On this basis, the negative correlations between motor impulsiveness and performance of judgment indexed with accuracy may reflect poor response inhibition for women who binge eat, which may make them vulnerable to binge eating.

Reaction times for incongruent trials in the flanker task were found to be significantly longer in women with BE, indicating a deficit of conflict monitoring in BE. The findings are comparable to the results of a study of combined electroencephalography (EEG) and eye tracking. In the food-related antisaccade task, Leehr et al. (2018) observed smaller N2 latencies in overweight individuals with BED compared with overweight individuals without BED, suggesting that the conflict processing might be less thorough in the overweight individuals with BED (Leehr et al., 2018). Our finding extends evidences of previous literature focusing on conflict monitoring in individuals with binge eat. In previous research, the Stroop color-word interference task was performed to assess the inhibitory control of individuals with eating disorders (Duchesne et al., 2010; Galioto et al., 2012; Balodis et al., 2013; Kittel et al., 2017). The mixed findings mentioned above could be explained by the methodological procedures employed. Corresponding to the differential mechanisms of the interference, the response of the flanker task and Stroop task may reflect different cognitive processes. For example, Tillman and Wiens (2011) assessed effects of variation in proportions of incongruent trials on response conflict in the Stroop and flanker task. ERP findings demonstrated that the flanker N200 and Stroop N450 may reflect different cognitive processes (Tillman and Wiens, 2011). The flanker N200 may reflect attentional control processes used to focus attention on task-relevant aspects of a situation, while the Stroop N450 may reflect the perceptual conflict processing (Tillman and Wiens, 2011). The flanker task contains two sources of conflict, one related to the responses, and the other related to the stimulus itself (Eriksen and Eriksen, 1974). The flankers involve little semantic interference, so the motor conflict may occur faster than does the conflict in the Stroop, which requires also semantic processing (Pires et al., 2014). Women who binge eat with high level of motor impulsiveness displayed longer reaction times in responding to high-caloric food images flanked by low-caloric food images (i.e., LHL) may reflect the poor inhibition of responses in the women who binge eat when exposure to food cues.

One alternative explanation from an evolutionary perspective is that the binge eating behavior may be suitably conceptualized as an “evolutionary mismatch” condition arising from a maladaptive gene–environment interaction (Levitan et al., 2004; Davis, 2015; Abed, 2016; Mayhew et al., 2018). The strong hedonic response to food was an undeniable survival benefit during the huntergatherer era, which mismatched to our current environment (Davis, 2014). The deficit of conflict monitoring in women who binge eat when food stimuli present may reflect an adaptative strategy in early environments.

A number of study limitations should be mentioned. First, given possible gender differences in BE (Rosenbaum and White, 2015), results do not necessarily apply to BE men. In addition, findings may not generalize fully to a wider population. Second, it would be beneficial to replicate these findings in food-related Stroop task. In these tasks, the food or eating words (e.g., cake, cream, and diet) are presented. Participants must name the color in which each word is printed and ignore the meaning of the words (Black et al., 1998; Johansson et al., 2005; Rosenbaum and White, 2015). Some studies have found that the Stroop effect is enhanced when food-related stimuli are primed in restrained eaters (Cooper and Fairburn, 1992; Perpiñá et al., 1993). Further, only high-caloric and low-caloric food stimuli were used, the conflict monitoring of food versus non-food stimuli cannot be revealed. Future research should examine the comparison of food versus non-food stimuli to investigate the food-specific conflict monitoring in the cognitive control processes. Finally, higher impulsive responding was correlated with more calories consumed in average weight samples (Nederkoorn et al., 2009). It would be interesting to investigate the correlation between the response conflict to high- and low-calorie foods and the consumption of these foods in future research.

## Conclusion

The results provide initial evidence that women who binge eat appear to exhibit greater response conflict deficits with food cues in a flanker task featured high-caloric food and low-caloric food images relative to those who do not have binge eating episodes. These findings suggest that individuals who binge eat tend to rely on automatic processing and respond to processing conflicting food stimuli less effectively. Such an understanding will assist to prevent disordered eating in binge-eating populations, and to develop psychological interventions more effectively.

## Ethics Statement

This study was carried out in accordance with the recommendations of the ethical guidelines of the American Psychological Association. The protocol was approved by the Human Research Ethics Committee of the Xinyang Normal University. All subjects gave written informed consent in accordance with the Declaration of Helsinki.

## Author Contributions

ZL designed the project. SL and MQ performed the experiment. ZL and PZ analyzed the data and wrote the manuscript.

## Conflict of Interest Statement

The authors declare that the research was conducted in the absence of any commercial or financial relationships that could be construed as a potential conflict of interest.
